# The quality of reporting general safety parameters and immune-related adverse events in clinical trials of FDA-approved immune checkpoint inhibitors

**DOI:** 10.1186/s12885-020-07518-5

**Published:** 2020-11-23

**Authors:** Zahra Karimian, Sandra Mavoungou, Joe-Elie Salem, Florence Tubach, Agnès Dechartres

**Affiliations:** 1 Sorbonne Université, Institut National de la Santé et de la Recherche Médicale (INSERM), Institut Pierre Louis d’Epidémiologie et de Santé Publique, AP-HP. Sorbonne Université, Hôpital Pitié Salpêtrière, Département de Santé Publique, Centre de Pharmacoépidémiologie de l’AP-HP (Cephepi), CIC-1422, F75013 Paris, France; 2 Sorbonne Université, Institut National de la Santé et de la Recherche Médicale (INSERM), Centre d’investigation clinique-1421, AP-HP. Sorbonne Université, Hôpital Pitié Salpêtrière, Départements de pharmacologie et cardiologie, UNICO-GRECO Cardio-Oncology program, F75013 Paris, France

**Keywords:** Randomized controlled trials, Immune checkpoint inhibitors, Reporting, Safety, Immune-related adverse events, Serious adverse events

## Abstract

**Background:**

While immune-checkpoint inhibitors (ICIs) have transformed the field of oncology for advanced-stage cancers, they can lead to serious immune toxicities. Several systematic reviews have evaluated the risk of immune-related adverse events (irAEs); however, most have focused on published articles without evaluating trial registries. The objective of this methodological review was to compare the quality of reporting of safety information and in particular, serious irAEs (irSAEs), in both publications and ClinicalTrials.gov for all current FDA-approved ICIs.

**Methods:**

PubMed was searched to retrieve all published phase III randomized controlled trials (RCTs) evaluating ICIs. For each eligible trial, we searched for corresponding registration on ClinicalTrials.gov and extracted relevant safety data from both the publication and results posted on registry. We then compared the quality of reporting and the value of safety data between both sources.

**Results:**

Of 42 eligible published trials, 34 had results posted on ClinicalTrials.gov. Considerable variability was noted in the reporting of safety in both sources. SAEs were reported for all trial results in ClinicalTrials.gov compared to 23.5% of publications. An overall incidence for irAEs and irSAEs was reported in 58.8 and 8.8% of publications respectively, compared to 11.8 and 5.9% in registry results. Comparing the value of specific irSAEs was not possible between the two sources in 32/34 trials either due to different reporting formats (61.8%) or data not being reported in one or both sources (32.4%). From the 2 studies with compatible irSAE format, only 1 had matching data in both sources.

**Conclusions:**

The reporting of irAEs / irSAEs varies considerably in publications and registries, which outlines the importance of standardizing the terminologies and methodologies for reporting safety information relevant to ICIs.

## Background

Immunotherapies have transformed the field of cancer therapy by improving the overall prognosis of patients, especially for recurrent and metastatic cancers [1–3]. Immune checkpoint inhibitors (ICIs) are a type of immunotherapy which result in increased activation of the immune system, allowing it to recognize and destroy tumor cells [4, 5]. However, ICI may also lead to potentially serious drug-induced immune toxicities collectively known as immune-related adverse events (irAEs), which, depending on their severity, may result in substantial declines in organ function and fatal outcomes [6, 7]. The rapid increase in the number of medication alerts regarding irAEs received by regulatory authorities suggests that immune toxicities may constitute a competing event with cancer evolution, making the assessment of irAE and serious irAEs (irSAE) a major concern. Despite efforts to develop standardized definitions and guidelines for their recognition and management [8], the reported incidence of irAEs varies greatly between studies ranging from 15 to 90% [9, 10], which may be partly due to inconsistent and incomplete reporting or characterization of AEs within clinical trials [11].

While previous systematic reviews have evaluated the quality of irAE reporting in publications of ICI clinical trials [12] or assessed their incidence [11], most have not considered information from clinical trial registries which include key information from the trial protocol (registered prior to patient recruitment), as well as results posted following trial completion (i.e., participant flow, primary and secondary endpoints and all serious and non-serious AEs). Registries are recognized as an important source of information when conducting systematic reviews, not only to identify unpublished trials and evaluate the risk of selective outcome reporting, but also to extract results, and in particular safety results [13, 14]. This is while previous studies have showed that certain safety information such as serious adverse events (SAEs) were more completely reported at ClinicalTrials.gov than in corresponding publications [15, 16].

Therefore, the primary objective of this study was to compare the quality of reporting of safety and irSAEs in particular between clinical trial publications and corresponding results posted on ClinicalTrials.gov for current FDA-approved immune checkpoint inhibitors (ICIs) in oncology.

## Methods

We performed a methodological review of the reporting of safety results and immune-related serious adverse events (irSAEs) in particular in publications and registries for all current FDA-approved ICIs (Additional file 1): CTLA-4 (ipilimumab), PD-1 (nivolumab, pembrolizumab) and PD-L1 (atezolizumab, avelumab, durvalumab and cemiplimab).

### Terminology and definitions

A complete and detailed list of the following terms and definitions which have been used in this study are provided in Additional file 2: Structural hierarchy of adverse events, severity of adverse events, seriousness of adverse events, immune-related adverse event (irAE), and immune-related serious adverse events (irSAE).

### Search for publications

A search in MEDLINE via PubMed was conducted to identify all randomized controlled trials (RCTs) assessing currently FDA-approved ICIs (Additional file 1). The search algorithm included key-words and free-text words for immune checkpoint inhibitor or blocker (anti-CTLA-4, anti-PD-1, anti-PD-L1) and drug names for currently FDA-approved ICIs and applied the Cochrane’s filter (sensitivity- and specificity-maximizing version) to identify RCTs (Additional file 3).

### Eligibility criteria

Phase III RCTs for all FDA-approved ICIs used in cancer treatment which were published in English prior to March 2019 were included in this study. Phase I, II or IV trials, duplicates, abstracts of conference proceedings, case reports / series, editorials, commentaries, expert opinions, letters, narrative reviews, secondary reports, retrospective analyses, systematic reviews and meta-analyses or non-English publications were excluded.

### Selection process

All references were evaluated for eligibility by one of the authors (ZK) with any doubtful publications considered upon evaluation and approval by a second author (AD). The screening procedure was conducted based on a two-step process: (1) title/abstract screening using Rayyan [17] and (2) full-text screening.

### Search for corresponding registration on ClinicalTrials.gov

For each selected published trial, ClinicalTrials.gov was searched for the corresponding RCT using the NCT number when provided in the publication. When the registration number was not reported (which was not the case for any of the eligible trials), we planned to search the trial acronym or key elements of the trial to identify the registration. According to the Food and Drug Administration Amendment Act of 2007 (FDAAA 801), applicable clinical trials (trials with at least one site in the US) must submit trial results within 12 months after the primary completion date. We therefore evaluated whether results were posted within 1 year for those concerned by the law.

### Data extraction

A structured data extraction form in Excel was used to collect the following information from publication and ClinicalTrials.gov for each trial, which was carried out in duplicate (ZK and SM) with any disagreements resolved through discussion and consensus:

#### From the published report

- Publication characteristics: title, first author, date of online publication, journal name, type of journal (specialty or general medical), funding source and whether the ClinicalTrial.gov NCT number was reported.
- Medical indications and interventions: type of cancer, stage, ICI medication administered, whether ICI was given as monotherapy or combination therapy and the treatment duration.
- Trial characteristics: study design, blinding (open label, single or double blind), countries where the trial was conducted, primary outcome (overall survival, progression-free survival, or other outcome), start and end dates of recruitment, sample size and planned follow-up duration.

#### From the registry results

- Registration information, trial start and primary completion dates, primary sponsor (pharmaceutical company, academic institution or other).

#### From both sources

- Evaluation of the reporting of safety: We evaluated the reporting of overall safety, and of irAEs and irSAEs from the text, tables and figures, as well as supplementary information (if any) using the following items based on the CONSORT Extension for Reporting Harms and safety guidelines / recommendations for reporting AEs in Oncology [18–20]:

##### Evaluation of general safety information

- Population of analysis: we evaluated whether safety was analyzed in all randomly assigned participants (intention-to-treat) or in a defined safety population (e.g., as-treated population) and we collected the number of participants analyzed in each treatment arm
- Use of a validated instrument for coding and grading AEs (MedDRA [21], CTCAE, etc.)
- Reporting of
  - a frequency threshold for AEs and SAEs (reporting of all AEs or only those occurring with a sufficient frequency)
  - the overall rate of AEs
  - the overall rate of SAEs
  - treatment-related adverse events (trAEs)
  - serious treatment-related adverse events (trSAEs)
  - withdrawals from treatment due to AEs and trAEs
  - death due to AE and trAEs

##### Evaluation of specific safety information associated with ICIs (irAEs and irSAEs)

- Terminology used: how irAEs were referred to (“select trAEs”, “AEs of interest”, “immune AEs”, “immune-related AEs” or “immune-mediated AEs”).
- Reporting of:
  - a definition for irAE and irSAEs
  - whether and how the investigators distinguished irAEs from other trAEs
  - an overall rate for irAEs and irSAEs
  - a frequency threshold for irAE and irSAEs (reporting of all AEs or only those occurring with a sufficient frequency)
  - structural hierarchy for description of irAE (MedRA System Organ Class (SOC) which is more general (e.g. skin, gastrointestinal) or Preferred Terms (PTs) which are more specific (e.g., rash, colitis), or any other level used for reporting irAEs)
  - the severity of irAE according to the NCI-CTCAE Grading Classification

Of note, AEs were considered as immune-related only when clearly indicated as such by the authors. In other words, similar trAEs as irAEs (e.g., pneumonitis or colitis) which did not have an underlying immune etiology were not considered in the assessment. Definitions for key terms are reported in Additional file 2.

### Comparison of key safety data between publications and registry results

For each trial, general safety parameters (listed above), as well as specific safety events associated with ICIs (irAEs and irSAEs), were compared between the publication and results posted on ClinicalTrials.gov. When this was not possible, the reason (unreported value, inconsistent reporting format, etc.) was noted.

We first extracted the incidence for each safety parameter from published trials and ClinicalTrials.gov for all trial arms and then compared the values by using the approach graphically illustrated in Additional file 4.

1. *Complete match*: when the reported values matched between the two sources for all treatment arms
2. *Partial match:* when the value matched for one / some arm(s), but not all arms of the trial
3. *Not a match*: if none of the reported values in the treatment arms matched between the two sources
4. *Not assessable or comparable:* if the value was not reported in one or both sources or if they were not presented using the same format

The reported frequencies from the two sources were marked as a *match*, if the rounded percentages were within ±1% of one another.

After comparing general safety information, we compared the overall incidence of irAEs and irSAEs, as well as the rates of specific types of irSAEs (e.g., pneumonitis, colitis, hepatitis, rash, etc.) between the two sources, for each trial arm. The same approach was used to compare the two sources (Additional file 4).

When there were several publications for a given trial, the article with a publication date *closer* to when the trial results were posted on ClinicalTrials.gov was considered for comparison. This was to ensure that any differences or discrepancies noted in the reported frequencies of key safety parameter between the two sources would not be attributable to updates in posting new trial results in the registry (basically, we wanted to make sure that detected differences were not a result of comparing newer trial results posted in ClinicalTrials.gov to old published information). Also, if the investigators of a trial had published efficacy and safety outcomes in separate articles, the publication reporting safety results was selected for the purposes of our study.

### Statistical analysis

Data analysis was descriptive. Frequencies and proportions are reported for categorical data, while median and interquartile ranges are presented for continuous data. Statistical analysis was performed using R software (v3.3.1).

## Results

### Selection process of published trials and general characteristics

**Fig. 1:**
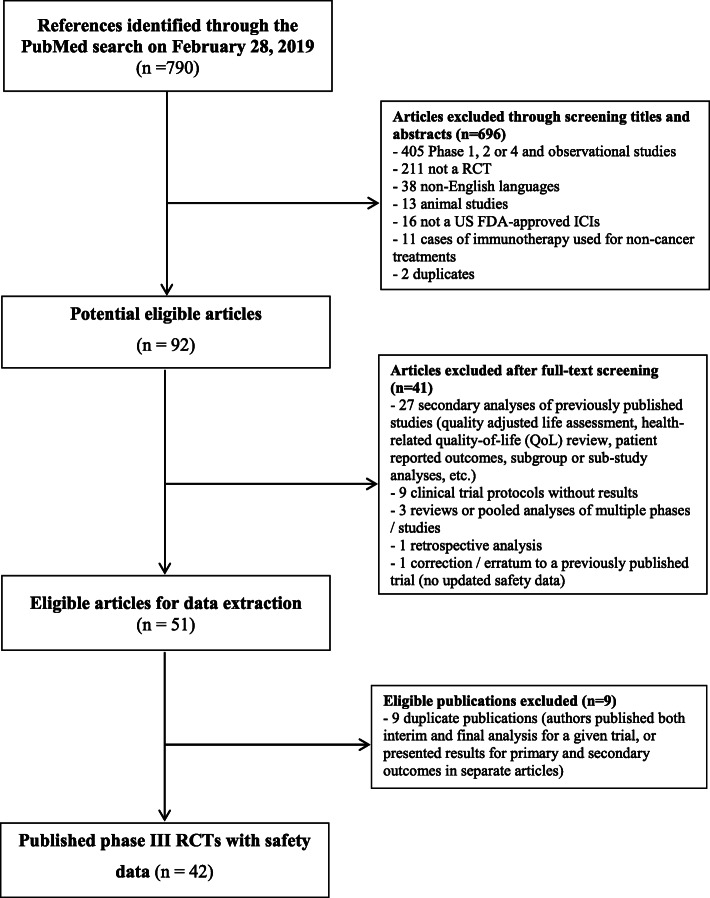
Study selection flowchart from PubMed search result

From the 790 references retrieved by the search, we identified 51 primary publications of phase III trials. An additional 9 references were excluded since some trials had multiple publications (Fig. 1). Of the 42 included trials published between August 2010 and February 2019 (Additional file 5), the most common indication was metastatic non-small cell lung cancer (*n* = 16, 38.1%). The median sample size was 695 (IQR 497–925). All trials had a parallel design, 36 (85.7%) had 2 study arms and 23 (54.8%) were conducted as open-label studies. The most common primary outcome was overall survival (*n* = 20, 47.6%) (Table 1).

**Table 1:** Characteristics of published phase III RCTs for current US FDA-approved Immune-Checkpoint Inhibitors (ICIs)

Published trials	***N*** = 42 ^**1**^
**Type of journal**	
Oncology	14 (33.3%)
General medicine	28 (66.7%)
**NCT number reported**	42 (100%)
**Immune-Checkpoint Inhibitors** ^**2**^	
Atezolizumab (Tecentriq®)	5 (11.9%)
Avelumab (Bavencio®)	3 (7.1%)
Cemiplimab (Libtayo®)	0 (0%)
Durvalumab (Imfinzi®)	2 (4.8%)
Ipilimumab (Yervoy®)	13 (31.0%)
Nivolumab (Opdivo®)	22 (33.3%)
Pembrolizumab (Keytruda®)	10 (23.8%)
**ICI regimen**	
Monotherapy with ICI	26 (61.9%)
Combination regimen of ICI with chemotherapy, radiotherapy, etc.	16 (38.1%)
**Medical indication**	
Metastatic non-small cell lung cancer (NSCLC)	16 (38.1%)
Unresectable or metastatic melanoma	11 (26.2%)
Renal cell carcinoma (RC)	4 (9.5%)
Gastroesophageal / gastric cancer (GEC/GC)	3 (7.1%)
Head and neck squamous cell carcinoma (HNSCC)	2 (4.8%)
Urothelial carcinoma (UC)	2 (4.8%)
Prostate cancer (PC)	2 (4.8%)
Breast cancer (BC)	1 (2.4%)
Small cell lung cancer (SCLC)	1 (2.4%)
**Study design**	
Parallel with 2 arms	36 (85.7%)
Parallel with 3 arms	6 (14.3%)
**Blinding**	
Open-label	23 (54.8%)
Double-blinded	19 (45.2%)
**Primary outcomes**	
Overall survival (OS)	20 (47.6%)
Progression Free Survival (PFS)	1 (2.4%)
Overall survival (OS) + Progression Free Survival (PFS)	14 (33.3%)
Recurrence Free Survival	3 (7.1%)
Safety outcomes	2 (4.8%)
Other (e.g., objective response rate, safety or other combinations)	2 (4.8%)
**RCT sites / countries**	
At least one site in the USA	37 (88.1%)
No site in the USA	5 (11.9%)
**Funding source**	
Pharmaceutical company	42 (100%)
Other (European Organization for Research and Treatment of Cancer)	1 (2.4%)

1. n (%), except otherwise indicated

2. The total percentages combined are more than 100% since 5 trials included both Ipilimumab and Nivolumab in one or more of their treatment arms

### Identification of corresponding trials in ClinicalTrials.gov and registration status

Of the 42 published RCTs, all were registered and the NCT number was systematically reported in the article, however, only 34 (81.0%) had results posted in the registry when we conducted our search on ClinicalTrials.gov (May 7, 2019). Of the 42 trials, 37 (88.1%) had at least one US site. Of these 37 trials, 18 (48.6%) had posted results within 1 year, 13 (35.1%) posted results after the deadline of 1 year and 6 (16.2%) currently do not have registry results posted (have not reached the deadline at this time).

### Evaluation of safety information

#### Overall or general safety information

- *Safety population:*

The population analyzed was indicated in all publications but was not clearly reported in 12 (35.2%) posts at ClinicalTrials.gov. All trials evaluated safety in patients who had taken at least one dose of the medication (as-treated population). The number of participants analyzed in each arm was reported in both sources and matched between the two sources in 32 (94.1%) trials.

b) *Use of standardized instruments for coding and grading AEs:*

All trials explicitly stated the use of MedDRA for coding AEs in their registry results compared to only 10 (29.4%) published trials. On the contrary, all publications noted using the NCI-CTCAE grading scale to report the severity of AEs, while only 4 (11.8%) trials reported similar grading of AEs in ClinicalTrials.gov.

c) *Reporting of safety parameters:*

**Fig. 2:**
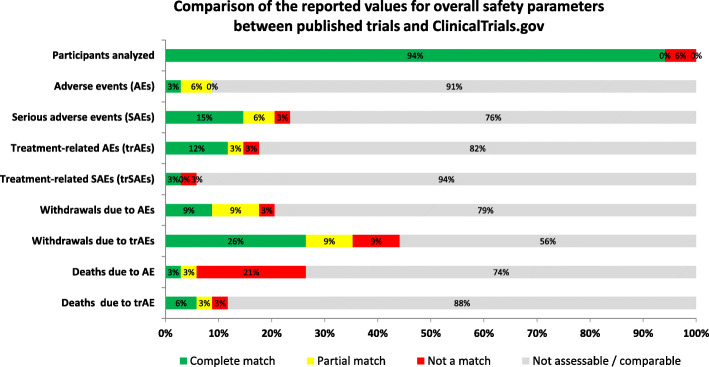
Comparison of general safety parameters in published RCTs of ICIs and corresponding registration at ClinicalTrials.gov. The order of the categories in each row from left to right: complete match, partial match, not a match, not comparable (due to results missing from CT.gov results, results missing in the published article, or results missing from both sources). The comparison of general safety indicators between the two sources (published trials and corresponding registration) is determined using the approach depicted in Additional file 4: Figure S1

In 36 (85.7%) publications, the authors did not report all AEs but only those reaching a threshold which varied across studies, ranging from events experienced by 2–3 patients to those encountered in at least 15% of participants. In ClinicalTrials.gov, a frequency threshold of 5% was used for reporting AEs in all trials while no threshold was used for reporting SAEs. The overall incidence of SAEs was reported in all 34 registry results whereas this information was reported in only 8 (23.5%) publications. More publications reported the overall incidence of AEs and trAEs (*n* = 17, 50.0% and *n* = 29, 85.3% respectively) compared to registry results (*n* = 3, 8.8% and *n* = 7, 20.6%). The number of deaths due to AEs was reported in 32 (94.1%) registry results compared to only 9 (26.5%) published trials. Of the 9 trials which had reported the number of deaths due to AEs in both the publication and ClinicalTrials.gov, the reported value did not match between the two sources in 7 (20.6%) out of the 9 trials (Fig. 2).

#### Immune-related adverse events (irAE)

- *Terminology and definitions:*

There was considerable variability in the terminology used for referring to irAE. Publications predominantly list them under immune-related AEs (*n* = 16, 38.11%), whereas most registry results refer to them as immune toxicities (*n* = 24, 70.6%). A clear definition for irAEs was provided in 35 (83.3%) of the 42 published trials compared to 4 (11.8%) trial results from the 34 RCTS with results posted on ClinicalTrials.gov, with even fewer trials defining irSAEs in publications (*n* = 19, 45.2%) and registry results (n = 1, 2.9%) respectively.

b) *Establishing drug causality for irAEs:*

All published trials which reported irAEs noted that drug-causality was adjudicated by the investigators and that they were labeled as immune-related regardless of whether the investigators attributed them to the treatment or not. Only 1 (2.9%) trial provided a distinction between immune-mediated AEs (imAEs) – AEs with an underlying immune mechanism not attributed to the ICI, and immune-related AEs (irAEs) – AEs with an immunogenic cause that were attributed to the ICI.

c) *Reporting of irAEs and irSAEs:*

**Fig. 3:**
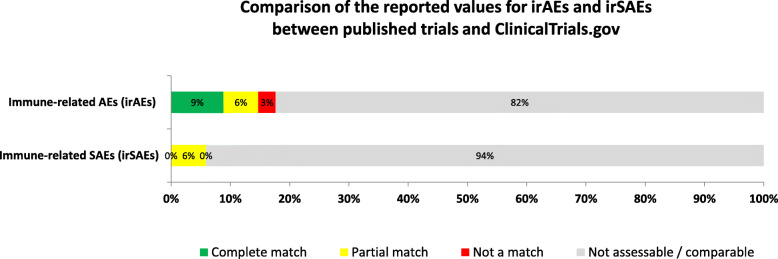
Comparison of irAEs and irSAEs between published RCTs of ICIs and corresponding registration at ClinicalTrials.gov. The order of the categories in each row from left to right: complete match, partial match, not a match, not comparable (due to results missing from CT.gov results, results missing in the published article, or results missing from both sources). The comparison of safety indicators specific to ICIs, immune-related AEs (irAEs) and serious immune-related AEs (irSAEs) between the two sources (published trials and corresponding registration) is determined using the approach depicted in Additional file 4: Figure S1

The overall incidence for irAEs and irSAEs were reported in 20 (58.8%) and 3 (8.8%) of publications respectively, compared to 4 (11.8%) and 2 (5.9%) of registry results (Fig. 3).

d) *Comparison of the incidence of specific types of irSAE between the two sources:*

**Fig. 4:**
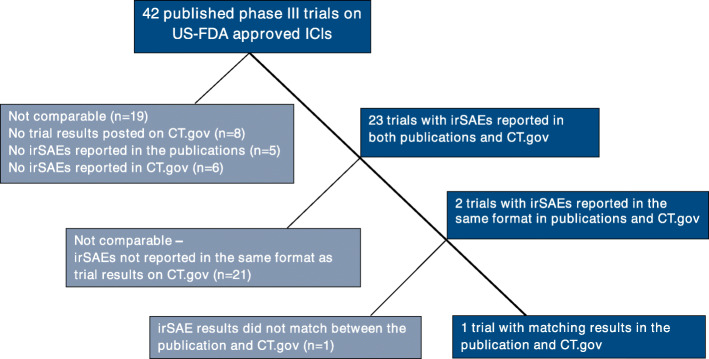
Comparison of the incidence of specific types of serious immune-related adverse events (irSAEs) between published RCTs and corresponding results posted on ClinicalTrials.gov (CT.gov)

For published trials, while the reporting format varied greatly depending on the level of structural hierarchy chosen by the authors – SOC, PT or both – all had indicated the use of the NCI-CTCAE grading scale for reporting the severity of irAEs. In contrast, all trial results posted on ClinicalTrials.gov reported the frequency of irAEs using PTs with only 2 (5.9%) of the 34 trials reporting using a grading scale for the severity of irAEs. Consequently, only 2 trials were identified as having a consistent reporting format to the registry for irSAEs, of which only 1 (2.9%) trial had matching results reported in both sources (Fig. 4).

Table 2 summarizes the differences in formatting components relevant to the reporting of safety data (including irSAEs) between publications and ClinicalTrials.gov.

**Table 2:** Differences in the Reporting Format of AEs (including irSAEs) between Published RCTs and Trial Results posted on ClinicalTrials.gov

Formatting component	Published trials	**ClinicalTrials.gov**
**Causality** Establishing drug-causality for AEs	Primarily report treatment-related AEs (trAEs)	All-cause AEs are reported regardless of drug causality
**Structural hierarchy** The level at which different types of AE are reported	System Organ Class (SOC) and / or Preferred Terms (PTs) according to MedDRA are used e.g., SOC: higher level group term (e.g., skin, GI) PT: lower level group term (e.g., rash, colitis)	Report AE occurrence using PTs, but typically not by SOCs
**Severity or grade** The intensity of an AE (mild, moderate, severe, etc.)	Often report AE grades; choice of presentation grading categories varies. e.g., some publications report grade 3–4 combined, others report grades 3, 4 and 5 together.	Grading is most often not reported in trial registry results
**Incidence of various types of AE** Reporting the number of patients or events	Generally report the number of AEs e.g., number of events which included rashes (including all grades and multiple episodes in patients, unless explicitly indicated that the highest grade per patient is reported)	Usually report the number of patients who experienced each specific type of AE e.g., number of patients in treatment arm 1 who experienced serious autoimmune colitis
**Frequency threshold** The incidence of AEs occurring beyond a certain threshold	Authors often choose a higher frequency threshold to report AEs in the main text, however, they may choose to report a more comprehensive list using a lower cutoff in the supplementary tables e.g., 10% in the main table and 1% in the supplement	ClinicalTrials.gov requires investigators to report all SAEs, and events ≥5% for non-SAEs

## Discussion

To our knowledge, this is the first study to provide a *comparative assessment* of the reporting of safety information (with a focus on irSAEs) for RCTs of ICIs between publications and registry results posted on ClinicalTrials.gov.

The major findings from our study were that: (1) key safety parameters were poorly reported in publications and ClinicalTrials.gov in particular irAE and irSAE despite their major importance; (2) even when certain safety parameters were reported in both sources, there was considerable variability in the reporting format (terminology) used for communicating this information, rendering a comparison difficult, and at times even impossible. The aforementioned prevented us from being able to present a comprehensive / global safety profile for each ICI.

The *inconsistencies* and *discrepancies* were notable in the reporting of both *general safety* (e.g., SAEs, deaths, etc.) and *specific safety* events associated with ICIs (irAE and irSAE), however, they were more extensive in the latter case. This considerable variability in *what and how* safety information was reported *across studies and between sources* is an impediment to pooling data and providing an accurate estimate for the frequency of key safety parameters from clinical trials investigating ICIs.

Similar to previous research, our study shows that certain safety results such as SAEs are more completely reported in ClinicalTrials.gov [15, 16, 22]. Since the reporting of all SAEs is mandatory in ClinicalTrials.gov, all trial results in the registry had reported SAEs while this key safety parameter was missing from 76.5% of published trials.

With regards to the overall incidence of irAEs and irSAEs specifically, they were reported in 58.8 and 8.8% of published trials respectively, as compared to 11.8 and 5.9% of trial results in the registry. An important consideration regarding the reporting of different types of irSAEs is that even though a breakdown of severe irAEs (grades 3–5) had been provided in all published trials, an overall incidence for irSAEs was not inferable / deducible. This is because while grade 3, 4 and 5 AEs are all considered serious by definition, SAEs do not only include events that are grades ≥3. Given that AEs grades < 3 (e.g., a grade 1 myocarditis or grade 2 rash) might occasionally require a medical intervention for symptomatic management or prevention from further progression, this will by definition result in their categorization as serious events. Therefore, the simple summation of the incidences of grades 3, 4 and 5 AEs would not accurately reflect the overall rate of irSAEs from an ICI. This underscores the importance of reporting the incidence of *both* severe and serious AEs for investigational drugs (including irAEs and irSAEs for ICIs).

The complete and accurate reporting of SAEs (including death and hospitalization), in particular irSAEs for ICIs – which are due to the drug’s mechanism of action – is crucial for this class of drug given their labeled indication is for metastatic and recurrent cancers who are prescribed the drug with increased survival in mind [23–29]. Furthermore, there has been an increasing number of safety alerts due to such events in recent years [6] which frequently include severe irAEs that can be fatal. While ICIs may improve survival outcomes in patients with advanced malignancies, a significant proportion of patients will not respond and still have a poor prognosis [30]. More importantly, considering end-of-life comfort and quality of life measures as well as avoiding substantial treatment-related costs play crucial roles in determining treatment goals in terminally ill cancer patients [31]. Therefore, a more comprehensive evaluation of the overall incidence and type of SAEs in particular irSAEs associated with these medications will allow terminal / end-stage cancer patients and their physicians to make more informed decisions by determining whether the benefits of increased survival outcomes will outweigh the risk of death and impaired quality of life due to toxicity from these drugs [32].

With regards to specific types of irSAE (e.g., pneumonitis, colitis), we were unable to compare their incidence between sources in 94.1% of trials, mainly because of differences in the reporting formats used for presenting safety data (61.8%). The most variable factors between the two sources were the structural hierarchy level used for reporting each type of irAE (e.g., the incidence of all irAEs affecting the skin compared to autoimmune dermatitis) and the choice to report grading for the severity of AEs (e.g., the rate of serious autoimmune colitis compared to grade 3 and 4 colitis). The variability in the terminology used for referring to this particular class of AEs further complicates matters when cross-checking their incidence. Our results showed that there were various ways of referring to the same AE in publications and registry results, which will need to be standardized. If indeed these terms refer to different AEs, the differences should be clearly explained by the authors. This is especially an important next step for the incorporation of ICIs as part of standard cancer treatment modalities [33, 34] since without the use of standardized terminologies and methods to consistently detect, collect, analyze and report irAEs [35, 36], efforts to provide accurate and reliable estimates for the rate of irSAEs of each ICI and cancer type remain hindered.

Finally, the findings of this research bear significant implications for the conduction of future systematic reviews and meta-analyses of irAEs and irSAE from ICIs. The evaluation of clinical trial registries is recommended by the Cochrane Handbook for Systematic Reviews of Interventions to limit the risk of publication bias and to extract results [13, 14]. The importance of ClinicalTrials.gov in facilitating the rapid understanding of harms for newer drugs such as ICIs had been recognized by previous studies [37], which is why it was selected as an information source for the extraction and comparison of safety data reported from ICI clinical trials in this study. Despite all this, from the numerous review articles on irAEs from ICIs, [15, 38–41] none have evaluated and compared safety results pertaining to irAEs from publications, clinical trial registries and regulatory documents so far. While ClinicalTrials.gov provides specific guidelines for the reporting of certain safety results such as SAEs for investigators, [42] it does not have a specific set of requirements or standardized reporting format for irAEs and irSAEs, making the extraction of this information difficult. Consequently, the inconsistencies in the reporting formats of irAEs and irSAEs remain an impediment to the incorporation of relevant safety data from all existing sources in systematic reviews and meta-analyses, which compromises the quality of the overall evidence on irAE and irSAE.

This study had some limitations. First, because of the inconsistency in irAE reporting format, we were unable to provide a robust comparative assessment of irSAE reporting from all currently published ICI trials (only 5.9% had a comparable format). Second, we did not consider regulatory documents from the FDA in our assessment (such as drug package inserts and review documents) for several reasons: (1) Since many metastatic cancers are considered as terminal and / or rare diseases, some new drug approvals by the FDA had been granted following phase II trials (pivotal trials) [43], which were excluded in the selection process of our study from the PubMed search results. (2) Another reason for only comparing published trials to results posted at ClinicalTrials.gov was that the FDA package inserts are regularly updated. Therefore, minimizing the effect of time as a variable on discrepancies noted between safety results extracted from three sources (publications, ClinicalTrials.gov and FDA package inserts) would have been even more far-fetched. (3) Finally, the FDA mandates no specific terminology for reporting AE data [37, 44], which if anything, would only further add to the discrepancies identified in the reporting of safety results for ICIs, had FDA package inserts been added to the comparison.

## Conclusion

This study highlights the insufficient and inconsistent reporting of key safety parameters, especially irAE and irSAEs, variability in terminology and discrepancies in the number of events in RCTs of ICIs. Comparability of safety information across trials and between various sources requires establishing a shared lexicon and mandating the reporting of key safety parameters. Adopting standardized terminology and consistency in the reporting methods of safety data in published trials and clinical trial registries is imperative; not only for the incorporation and pooling of safety information from all existing sources, and providing better estimates for the incidence of AEs in systematic reviews and meta-analyses, but ultimately for transparent communication in medical practice.

## Supplementary information

**Additional file 1: Appendix 1.** Current US-FDA approved Immune Checkpoint Inhibitors (ICIs) and labeled indications (April 2019).

Additional file 2: **Appendix 2.** Terminologies and definitions used for reporting safety results in ICI RCTs.

Additional file 3: **Appendix 3.** PubMed search algorithm.PubMed search algorithm.

Additional file 4: **Appendix 4. Figure S1.** Comparison of the incidence of safety data reported in publications and corresponding registry results from ClinicalTrials.gov.

Additional file 5: **Appendix 5.** Complete list of published phase III RCTs of ICIs included in the analysis.

## Data Availability

The datasets used and/or analyzed in this study are available from the corresponding author upon reasonable request.

## Abbreviations

AE: Adverse event
CTCAE: Common terminology criteria for adverse events
ICH: International conference on harmonisation
ICI: Immune-checkpoint inhibitor
irAE: Immune-related adverse event
irSAE: Serious immune-related adverse event
MedDRA: Medical dictionary for regulatory activities
NCI: National cancer institute
PT: Preferred term
RCT: Randomized controlled trial
SAE: Serious adverse event
SOC: System organ class
trAE: Treatment-related adverse event
trSAE: Serious treatment-related adverse event
US-FDA: United States food and drug administration
