# Supplementary material for: The quality of reporting general safety parameters and immune-related adverse events in clinical trials of FDA-approved immune checkpoint inhibitors
Source: BMC Cancer. 2020 Nov 23;20:1128. doi: 10.1186/s12885-020-07518-5 (PMC7682068; doi:10.1186/s12885-020-07518-5)
Supplement: Supplementary file 1 — Additional file 1: Appendix 1. Current US-FDA approved Immune Checkpoint Inhibitors (ICIs) and labeled indications (April 2019). [file 12885_2020_7518_MOESM1_ESM.docx]

**Appendix 1 – Current US-FDA approved Immune Checkpoint Inhibitors (ICIs) and labeled indications (April 2019)**

| **ICI generic name**  **(Brand / trade name)** | **Manufacturer** | **Inhibitor target on T-Cell or cancer cells** | **First approval**  **date by the FDA** | **Medical indications**  **(Recurrent and metastatic cancers) ^1^** |
| --- | --- | --- | --- | --- |
| Ipilimumab (Yervoy®) | Bristol-Myers Squibb Co. | CTLA-4 / T-Cell | 2011 | Unresectable or metastatic melanoma |
| Nivolumab (Opdivo®) | Bristol-Myers Squibb Co. | PD-1 / T-Cell | 2014 | Squamous and non-squamous NSCLC, RCC, cHL, HNSCC , UC, CRC , HCC |
| Atezolizumab (Tecentriq®) | La Roche / Genentech Inc. | PD-L1 / cancer cell | 2016 | UC, NSCLC |
| Pembrolizumab (Keytruda®) | Merck and Co. Inc. | PD-1 / T-Cell | 2017 | Adult / pediatric refractory cHL , UC, adult / pediatric unresectable MSI-H or dMMR solid tumors or MSI-H or dMMR CRC, GC/GEJ adenocarcinoma |
| Avelumab (Bavencio®) | Merck and Co. Inc. / Pfizer | PD-L1 / cancer cell | 2017 | Metastatic Merkel cell carcinoma |
| Durvalumab (Imfinzi®) | AstraZeneca UK Ltd. | PD-L1 / cancer cell | 2017 | UC, unresectable stage III NSCLC |
| Cemiplimab (*Libtayo®*) | Sanofi | PD-L1 / cancer cell | 2018 | CSCC |
| Tremelimumab | AstraZeneca UK Ltd. | CTLA-4 / T-Cell | Not yet approved | Mesothelioma |

**Abbreviations:**

Head and neck squamous cell carcinoma (HNSCC)

Cutaneous squamous cell carcinoma (CSCC)

Metastatic non-small cell lung cancer (NSCLC)

Small cell lung cancer (SCLC)

Hepatocellular carcinoma (HCC)

Gastroesophageal junction */* gastric cancer (GEJ/GC)

Microsatellite instability-high (MSI-H) or mismatch repair deficient (dMMR) metastatic colorectal cancer (CRC)

Urothelial carcinoma (UC)

Renal cell carcinoma (RC)

Breast cancer (BC)

Prostate cancer (PC)

Classical Hodgkin lymphoma (cHL)
