# Supplementary material for: The quality of reporting general safety parameters and immune-related adverse events in clinical trials of FDA-approved immune checkpoint inhibitors
Source: BMC Cancer. 2020 Nov 23;20:1128. doi: 10.1186/s12885-020-07518-5 (PMC7682068; doi:10.1186/s12885-020-07518-5)
Supplement: Supplementary file 2 — Appendix 2. Terminologies and definitions used for reporting safety results in ICI RCTs. [file 12885_2020_7518_MOESM2_ESM.docx]

**Appendix 2 – Terminologies and definitions used for reporting safety results in ICI RCTs**

**Structural hierarchy of adverse events** – The Medical Dictionary for Regulatory Activities (MedDRA) developed by the International Conference on Harmonisation of Technical Requirements for Registration of Pharmaceuticals for Human Use (ICH) is a clinically-validated and standardized medical terminology used by regulatory authorities and the biopharmaceutical industry intended to facilitate the sharing of information regarding pharmaceuticals, vaccines and drug-device combination products internationally. The various types of AEs can be reported using MedDRA at any of the general or specific levels listed below:

1. System Organ Class (SOC) – most general level

e.g., Skin, Gastrointestinal System

1. High Level Group Term (HLGT)
2. High Level Term (HLT)
3. Preferred Term (PT)

e.g., Rash, Colitis

1. Lowest Level Term (LLT) – most specific level

**Severity of adverse events (AEs) –** The National Cancer Institute’s (NCI) Common Terminology Criteria for Adverse Events (CTCAE) provides a grading scale that is utilized for AE reporting of oncology drugs. According to the NCI-CTCAE:

Grade 1: mild – asymptomatic or mild symptoms; observation only, intervention not indicated

Grade 2: moderate – minimal or non-invasive intervention indicated

Grade 3: severe – medically significant but not immediately life-threatening; hospitalization or prolongation of hospitalization indicated

Grade 4: very severe – life-threatening consequences; urgent intervention indicated

Grade 5: death related to AE or fatal AEs

**Seriousness of adverse drug events (SAEs)** – SAEs are defined as an untoward medical occurrence associated with the use of a medical product which results in death, permanent injury, significant disability/incapacity, congenital anomaly, requiring hospitalization (initial or prolonged) or intervention to prevent permanent impairment/damage.

**Immune-related adverse events (irAEs)** – irAEs are a unique class of AEs associated with ICIs which are described according to the signs and symptoms affecting various organ systems and graded according to the severity of symptoms. The characterization of AEs as immune-related requires that they be possibly related to the study drug (treatment-related) and to be included in a preassembled / predefined list of inflammatory AEs consistent with an autoimmune etiology or mechanism.

**Serious immune-related adverse events (irSAEs)** – Based on the NCI-CTCAE classification system and descriptions for each grade category provided above, Grade 3 to 5 irAEs would be considered as serious AEs. However, not all irSAEs are those with Grades ≥ 3. Basically, irAEs of lower Grades (1 – 2) can be classified as serious depending on the outcome of the event.

Finally, the management of irAEs generally include administration of corticosteroids (orally or intravenously), a delay in a scheduled dose, or discontinuation of ICI therapy altogether. According to the American Society for Clinical Oncology (ASCO) and European Society for Medical Oncology (ESMO) guidelines, Grade 3 and 4 irAEs require administration of high dose corticosteroids for symptom relief, while Grade 3 irAEs warrant holding the medication, and Grade 4 irAEs generally require permanent discontinuation of ICI therapy. Therefore, the management guidelines for severe irAEs (Grades ≥ 3) reinforce their classification as serious AEs.
