# Supplementary material for: The quality of reporting general safety parameters and immune-related adverse events in clinical trials of FDA-approved immune checkpoint inhibitors
Source: BMC Cancer. 2020 Nov 23;20:1128. doi: 10.1186/s12885-020-07518-5 (PMC7682068; doi:10.1186/s12885-020-07518-5)
Supplement: Supplementary file 3 — Appendix 3. PubMed search algorithm.PubMed search algorithm. [file 12885_2020_7518_MOESM3_ESM.docx]

**Appendix 3 – PubMed search algorithm**

**Identification of phase III Randomized Controlled Trials (RCTs) of currently US-FDA approved Immune Checkpoint Inhibitors (ICIs) using Cochrane’s filter (sensitivity- and specificity-maximizing version):**

1. "immune checkpoint inhibitor*”[title]
2. "immune checkpoint blocker*"[title]
3. “cytotoxic T-lymphocyte associated antigen-4”[title]
4. “programmed cell death 1 receptor”[title]
5. “programmed cell death-Ligand 1”[title]
6. CTLA-4[title]
7. PD-1[title]
8. PD-L1[title]
9. anti-CTLA-4[title]
10. anti-PD-1[title]
11. anti-PD-L1[title]
12. ipilimumab[mh]
13. ipilimumab[title]
14. Yervoy[title]
15. nivolumab[mh]
16. nivolumab[title]
17. Opdivo[title]
18. atezolizumab[title]
19. Tecentriq[title]
20. pembrolizumab[title]
21. Keytruda[title]
22. avelumab[title]
23. Bavencio[title]
24. durvalumab[title]
25. Cemiplimab[title]
26. #1 OR #2 OR #3 OR #4 OR #5 OR #6 OR #7 OR #8 OR #9 OR #10 OR #11 OR #12 OR #13 OR #14 OR #15 OR #16 OR #17 OR #18 OR #19 OR #20 OR #21 OR #22 OR #23 OR #24 OR #25 🡪 n=10020

**Identifying RCTs in PubMed with Cochrane’s filter (sensitivity- and precision-maximizing version)**

27. randomized controlled trial[pt]

28. controlled clinical trial[pt]

29. randomized[tiab]

30. placebo[tiab]

31. clinical trials as topic[mesh:noexp]

32. randomly[tiab]

33. trial[ti]

34. #27 OR #28 OR #29 OR #30 OR #31 OR #32 OR #33

35. animals[mh] NOT humans [mh]

36. #34 NOT #35 🡪 n=1112394

37. #26 AND #36 🡪 **n=790**
