# Supplementary material for: The quality of reporting general safety parameters and immune-related adverse events in clinical trials of FDA-approved immune checkpoint inhibitors
Source: BMC Cancer. 2020 Nov 23;20:1128. doi: 10.1186/s12885-020-07518-5 (PMC7682068; doi:10.1186/s12885-020-07518-5)
Supplement: Supplementary file 4 — Appendix 4. Figure S1. Comparison of the incidence of safety data reported in publications and corresponding registry results from ClinicalTrials.gov. [file 12885_2020_7518_MOESM4_ESM.docx]

**Appendix 4 – Fig. S1 Comparison of the incidence of safety data reported in publications and corresponding registry results from ClinicalTrials.gov**

**
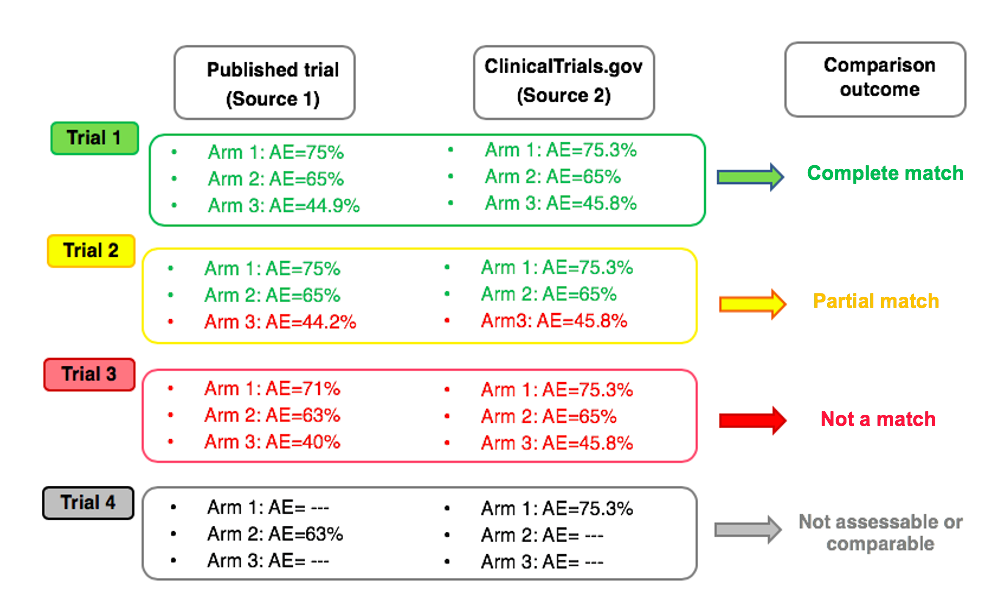
**

Frequencies were considered to match between the two sources if the rounded percentages were within (± 1%) of each other
