# Supplementary material for: The quality of reporting general safety parameters and immune-related adverse events in clinical trials of FDA-approved immune checkpoint inhibitors
Source: BMC Cancer. 2020 Nov 23;20:1128. doi: 10.1186/s12885-020-07518-5 (PMC7682068; doi:10.1186/s12885-020-07518-5)
Supplement: Supplementary file 5 — Appendix 5. Complete list of published phase III RCTs of ICIs included in the analysis. [file 12885_2020_7518_MOESM5_ESM.docx]

**Appendix 5 – Complete list of published phase III RCTs of ICIs included in the analysis**

1. Antonia, S. J. *et al.* Overall Survival with Durvalumab after Chemoradiotherapy in Stage III NSCLC. *New England Journal of Medicine* 379, 2342–2350 (2018).

2. Ascierto, P. A. *et al.* Ipilimumab 10 mg/kg versus ipilimumab 3 mg/kg in patients with unresectable or metastatic melanoma: a randomised, double-blind, multicentre, phase 3 trial. *The Lancet Oncology* 18, 611–622 (2017).

3. Bang, Y.-J. *et al.* Phase III, randomised trial of avelumab versus physician’s choice of chemotherapy as third-line treatment of patients with advanced gastric or gastro-oesophageal junction cancer: primary analysis of JAVELIN Gastric 300. *Annals of Oncology* 29, 2052–2060 (2018).

4. Barlesi, F. *et al.* Avelumab versus docetaxel in patients with platinum-treated advanced non-small-cell lung cancer (JAVELIN Lung 200): an open-label, randomised, phase 3 study. *The Lancet Oncology* 19, 1468–1479 (2018).

5. Beer, T. M. *et al.* Randomized, Double-Blind, Phase III Trial of Ipilimumab Versus Placebo in Asymptomatic or Minimally Symptomatic Patients With Metastatic Chemotherapy-Naive Castration-Resistant Prostate Cancer. *Journal of Clinical Oncology* 35, 40–47 (2017).

6. Bellmunt, J. *et al.* Pembrolizumab as Second-Line Therapy for Advanced Urothelial Carcinoma. *New England Journal of Medicine* 376, 1015–1026 (2017).

7. Borghaei, H. *et al.* Nivolumab versus Docetaxel in Advanced Nonsquamous Non–Small-Cell Lung Cancer. *New England Journal of Medicine* 373, 1627–1639 (2015).

8. Brahmer, J. *et al.* Nivolumab versus Docetaxel in Advanced Squamous-Cell Non–Small-Cell Lung Cancer. *New England Journal of Medicine* 373, 123–135 (2015).

9. Carbone, D. P. *et al.* First-Line Nivolumab in Stage IV or Recurrent Non–Small-Cell Lung Cancer. *New England Journal of Medicine* 376, 2415–2426 (2017).

10. Chih-Hsin Yang, J. *et al.* Osimertinib Plus Durvalumab versus Osimertinib Monotherapy in EGFR T790M–Positive NSCLC following Previous EGFR TKI Therapy: CAURAL Brief Report. *Journal of Thoracic Oncology* 14, 933–939 (2019).

11. Cohen, E. E. W. *et al.* Pembrolizumab versus methotrexate, docetaxel, or cetuximab for recurrent or metastatic head-and-neck squamous cell carcinoma (KEYNOTE-040): a randomised, open-label, phase 3 study. *The Lancet* 393, 156–167 (2019).

12. Eggermont, A. M. M. *et al.* Adjuvant Pembrolizumab versus Placebo in Resected Stage III Melanoma. *New England Journal of Medicine* 378, 1789–1801 (2018).

13. Eggermont, A. M. M. *et al.* Prolonged Survival in Stage III Melanoma with Ipilimumab Adjuvant Therapy. *New England Journal of Medicine* 375, 1845–1855 (2016).

14. Fehrenbacher, L. *et al.* Updated Efficacy Analysis Including Secondary Population Results for OAK: A Randomized Phase III Study of Atezolizumab versus Docetaxel in Patients with Previously Treated Advanced Non–Small Cell Lung Cancer. *Journal of Thoracic Oncology* 13, 1156–1170 (2018).

15. Ferris, R. L. *et al.* Nivolumab vs investigator’s choice in recurrent or metastatic squamous cell carcinoma of the head and neck: 2-year long-term survival update of CheckMate 141 with analyses by tumor PD-L1 expression. *Oral Oncology* 81, 45–51 (2018).

16. Gandhi, L. *et al.* Pembrolizumab plus Chemotherapy in Metastatic Non–Small-Cell Lung Cancer. *New England Journal of Medicine* 378, 2078–2092 (2018).

17. Govindan, R. *et al.* Phase III Trial of Ipilimumab Combined With Paclitaxel and Carboplatin in Advanced Squamous Non–Small-Cell Lung Cancer. *Journal of Clinical Oncology* 35, 3449–3457 (2017).

18. Hellmann, M. D. *et al.* Nivolumab plus Ipilimumab in Lung Cancer with a High Tumor Mutational Burden. *New England Journal of Medicine* 378, 2093–2104 (2018). *

19. Herbst, R. S. *et al.* Pembrolizumab versus docetaxel for previously treated, PD-L1-positive, advanced non-small-cell lung cancer (KEYNOTE-010): a randomised controlled trial. *The Lancet* 387, 1540–1550 (2016).

20. Hodi, F. S. *et al.* Improved Survival with Ipilimumab in Patients with Metastatic Melanoma. *New England Journal of Medicine* 363, 711–723 (2010).

21. Horn, L. *et al.* First-Line Atezolizumab plus Chemotherapy in Extensive-Stage Small-Cell Lung Cancer. *New England Journal of Medicine* 379, 2220–2229 (2018). *

22. Kang, Y.-K. *et al.* Nivolumab in patients with advanced gastric or gastro-oesophageal junction cancer refractory to, or intolerant of, at least two previous chemotherapy regimens (ONO-4538-12, ATTRACTION-2): a randomised, double-blind, placebo-controlled, phase 3 trial. *The Lancet* 390, 2461–2471 (2017). *

23. Kwon, E. D. *et al.* Ipilimumab versus placebo after radiotherapy in patients with metastatic castration-resistant prostate cancer that had progressed after docetaxel chemotherapy (CA184-043): a multicentre, randomised, double-blind, phase 3 trial. *The Lancet Oncology* 15, 700–712 (2014).

24. Larkin, J. *et al.* Combined Nivolumab and Ipilimumab or Monotherapy in Untreated Melanoma. *New England Journal of Medicine* 373, 23–34 (2015).

25. Larkin, J. *et al.* Overall Survival in Patients With Advanced Melanoma Who Received Nivolumab Versus Investigator’s Choice Chemotherapy in CheckMate 037: A Randomized, Controlled, Open-Label Phase III Trial. *Journal of Clinical Oncology* 36, 383–390 (2018).

26. Lebbé, C. *et al.* Evaluation of Two Dosing Regimens for Nivolumab in Combination With Ipilimumab in Patients With Advanced Melanoma: Results From the Phase IIIb/IV CheckMate 511 Trial. *Journal of Clinical Oncology* 37, 867–875 (2019).

27. Motzer, R. J. *et al.* Nivolumab versus Everolimus in Advanced Renal-Cell Carcinoma. *New England Journal of Medicine* 373, 1803–1813 (2015).

28. Motzer, R. J. *et al.* Avelumab plus Axitinib versus Sunitinib for Advanced Renal-Cell Carcinoma. *New England Journal of Medicine* 380, 1103–1115 (2019). *

29. Motzer, R. J. *et al.* Nivolumab plus Ipilimumab versus Sunitinib in Advanced Renal-Cell Carcinoma. *New England Journal of Medicine* 378, 1277–1290 (2018).

30. Paz-Ares, L. *et al.* Pembrolizumab plus Chemotherapy for Squamous Non–Small-Cell Lung Cancer. *New England Journal of Medicine* 379, 2040–2051 (2018).

31. Powles, T. *et al.* Atezolizumab versus chemotherapy in patients with platinum-treated locally advanced or metastatic urothelial carcinoma (IMvigor211): a multicentre, open-label, phase 3 randomised controlled trial. *The Lancet* 391, 748–757 (2018).

32. Reck, M. *et al.* Phase III Randomized Trial of Ipilimumab Plus Etoposide and Platinum Versus Placebo Plus Etoposide and Platinum in Extensive-Stage Small-Cell Lung Cancer. *Journal of Clinical Oncology* 34, 3740–3748 (2016).

33. Reck, M. *et al.* Pembrolizumab versus Chemotherapy for PD-L1–Positive Non–Small-Cell Lung Cancer. *New England Journal of Medicine* 375, 1823–1833 (2016).

34. Rini, B. I. *et al.* Pembrolizumab plus Axitinib versus Sunitinib for Advanced Renal-Cell Carcinoma. *New England Journal of Medicine* 380, 1116–1127 (2019). *

35. Robert, C. *et al.* Nivolumab in Previously Untreated Melanoma without *BRAF* Mutation. *New England Journal of Medicine* 372, 320–330 (2015).

36. Robert, C. *et al.* Ipilimumab plus Dacarbazine for Previously Untreated Metastatic Melanoma. *New England Journal of Medicine* 364, 2517–2526 (2011).

37. Schachter, J. *et al.* Pembrolizumab versus ipilimumab for advanced melanoma: final overall survival results of a multicentre, randomised, open-label phase 3 study (KEYNOTE-006). *The Lancet* 390, 1853–1862 (2017).

38. Schmid, P. *et al.* Atezolizumab and Nab-Paclitaxel in Advanced Triple-Negative Breast Cancer. *New England Journal of Medicine* 379, 2108–2121 (2018). *

39. Shitara, K. *et al.* Pembrolizumab versus paclitaxel for previously treated, advanced gastric or gastro-oesophageal junction cancer (KEYNOTE-061): a randomised, open-label, controlled, phase 3 trial. *The Lancet* 392, 123–133 (2018).

40. Socinski, M. A. *et al.* Atezolizumab for First-Line Treatment of Metastatic Nonsquamous NSCLC. *New England Journal of Medicine* 378, 2288–2301 (2018). *

41. Weber, J. *et al.* Adjuvant Nivolumab versus Ipilimumab in Resected Stage III or IV Melanoma. *New England Journal of Medicine* 377, 1824–1835 (2017). *

42. Wu, Y.-L. *et al.* Nivolumab Versus Docetaxel in a Predominantly Chinese Patient Population With Previously Treated Advanced NSCLC: CheckMate 078 Randomized Phase III Clinical Trial. *Journal of Thoracic Oncology* 14, 867–875 (2019).

** Published trials without results posted on ClinicalTrials.gov at the time of analysis (May 7, 2019)*
